# Regulation of Functional Protein Aggregation by Multiple Factors: Implications for the Amyloidogenic Behavior of the CAP Superfamily Proteins

**DOI:** 10.3390/ijms21186530

**Published:** 2020-09-07

**Authors:** Jie Sheng, Nick K. Olrichs, Bart M. Gadella, Dora V. Kaloyanova, J. Bernd Helms

**Affiliations:** Department of Biomolecular Health Sciences, Faculty of Veterinary Medicine, Utrecht University, 3584 CM Utrecht, The Netherlands; J.Sheng@uu.nl (J.S.); nolrichs@hotmail.com (N.K.O.); B.M.Gadella@uu.nl (B.M.G.); D.V.Kaloyanova@uu.nl (D.V.K.)

**Keywords:** protein aggregation, functional amyloids, amyloids, polyanions, heparin, metal ions, GAPR-1, CAP superfamily

## Abstract

The idea that amyloid fibrils and other types of protein aggregates are toxic for cells has been challenged by the discovery of a variety of functional aggregates. However, an identification of crucial differences between pathological and functional aggregation remains to be explored. Functional protein aggregation is often reversible by nature in order to respond properly to changing physiological conditions of the cell. In addition, increasing evidence indicates that fast fibril growth is a feature of functional amyloids, providing protection against the long-term existence of potentially toxic oligomeric intermediates. It is becoming clear that functional protein aggregation is a complexly organized process that can be mediated by a multitude of biomolecular factors. In this overview, we discuss the roles of diverse biomolecules, such as lipids/membranes, glycosaminoglycans, nucleic acids and metal ions, in regulating functional protein aggregation. Our studies on the protein GAPR-1 revealed that several of these factors influence the amyloidogenic properties of this protein. These observations suggest that GAPR-1, as well as the cysteine-rich secretory proteins, antigen 5 and pathogenesis-related proteins group 1 (CAP) superfamily of proteins that it belongs to, require the assembly into an amyloid state to exert several of their functions. A better understanding of functional aggregate formation may also help in the prevention and treatment of amyloid-related diseases.

## 1. Introduction to Protein Aggregates: Two Sides of a Coin

Protein aggregation is a biochemical process in which proteins accumulate or clump together to form aggregates and/or membrane-less inclusions, either intracellularly or extracellularly [1,2,3,4]. For a long time, protein aggregation was viewed exclusively as a pathological process, due to its intimate relation with a number of devastating diseases. Amyloid formation is a distinct type of protein aggregation in which soluble proteins assemble into highly ordered, biochemically stable fibrils with a cross-β structure [3,5,6]. To date, approximately 50 amyloid-forming peptides and proteins have been identified as the pathological hallmark of many human disorders [4]. However, the notion that protein aggregation is inherently detrimental to cells has recently been challenged by the identification of protein aggregates that play functional roles in physiology. The structure of amyloids enables their use as scaffolds for biochemical activities and the compact nature of aggregates makes them highly suitable as sites for protein storage. Functional protein aggregates appeared crucial for a variety of biological activities, including the storage of peptide hormones [7,8], reproduction and fertilization [9,10,11], pigmentation [12], necroptosis [13], antimicrobial responses [14], adaptation to stress [15,16], cellular dormancy [17,18,19], bacterial biofilm formation [20,21], regulating fungal-host and fungal-fungal interactions [22], modulating epigenetic heritable phenotypes in yeast [23] and the persistence of long-term memory in *Drosophila* [24].

**Figure 1:**
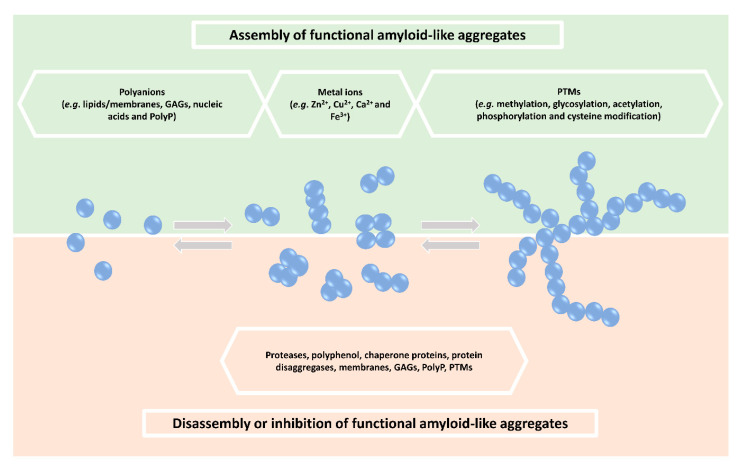
Factors modulating the dynamics of functional amyloid-like aggregates. Factors involved in regulating assembly of functional amyloid-like aggregates (top half) and/or disassembly to inhibit pathological amyloid-like aggregates (bottom half) are indicated in the figure.

The double-sided nature of amyloids as pathological or physiological assemblies, together with several shared similarities (e.g., β-sheet structure, thermodynamic stability, specific tinctorial properties [25], resistance to proteases, heat and SDS treatment, and similarities in high-resolution structures [26,27]), make it challenging to clearly distinguish between “bad” and “good” amyloid aggregates. To address this, a better characterization of the nature of protein aggregates will be required by the consideration of additional properties, including function, reversibility, infectivity, localization, composition and structure [3]. Factors affecting the amyloidogenic properties of proteins are summarized in Figure 1 and will be discussed hereafter.

## 2. Regulation of Functional Amyloid-Like Aggregate Formation

In contrast to pathological aggregates, functional aggregates are assembled under physiological conditions without deleterious effects on cells, suggesting that the formation of these two types of aggregates are regulated by different mechanisms. Soluble oligomeric intermediates formed during aberrant amyloid aggregation are detrimental to cells [28,29]. The presence of these oligomers in cells can result in membrane permeabilization, elevated Ca^2+^ concentrations, oxidative stress and cell death [28,29,30,31]. Although functional aggregates are not lethal to cells, potentially toxic intermediates do exist during their assembly [32]. For example, the intermediate oligomers of pigment cell-specific pre-melanosomal protein (PMEL) and Orb2, a key protein for long-term memory formation in *Drosophila*, are toxic, whereas the mature amyloid state of these proteins is functional [33,34]. In order to avoid toxicity, functional aggregation must therefore be tightly regulated [32].

### 2.1. Kinetics

Different from pathological aggregates, functional aggregation processes often occur with relatively fast kinetics. Rapid growth rates of amyloid fibrils have been proposed as one of the protective mechanisms to minimize the existence of intermediate oligomers [32,34]. For instance, recombinant PMEL exhibits rapid fibrilization in vitro, with a fast transition from the monomeric state to mature amyloids. Unlike pathological amyloids, which typically need several days to be formed in vitro, the PMEL fibrillogenic domain requires only several minutes to form fibrils [12,35,36]. A similar rapid fibrillation is observed for other functional amyloids, such as the antimicrobial peptide protegrin-1 (PG-1), cystatin-related epididymal spermatogenic (CRES) proteins, Orb2, and the bacterial curli protein CsgA [11,14,34,37]. CsgA monomers form nascent fibrils very fast under native conditions, directly folding and oligomerizing into minimal fibers and bypassing a transition through an intermediate, non-amyloid oligomeric state [37]. When the prion-like domain (PLD) of Orb2 is swapped with the amyloidogenic polyglutamine tract of exon 1 of the human huntingtin protein, the chimeric Orb2 protein assembled much slower and with a longer existence of highly toxic oligomeric states. This suggests that, in principle, Orb2 can form a toxic conformation. Vice versa, when the amyloidogenic polyglutamine tract of exon 1 of the human huntingtin protein is replaced with the PLD of Orb2, the chimeric huntingtin construct formed nontoxic, short-lived species. Altogether, rapid growth rate seems to be one of the features which can distinguish functional from pathological aggregates [38].

### 2.2. Reversibility

As indicated above, protein aggregation has been widely used to control multiple physiological processes in cells. Functional protein aggregation is often characterized by the reversible nature of the aggregation process. Firstly, upon exposure to stress (e.g., heat shock, acidosis, nutrient starvation, etc.), cells can assemble different types of condensates, such as nuclear amyloid bodies (A-bodies) [16], Balbiani bodies [18], processing bodies (P-bodies) [39], heat-shock granules (HSGs) [40] and cytoplasmic foci that contain heterogeneous protein:RNA complexes with amyloid-like biophysical properties [15,41]. A well-studied example is the reversible formation of functional amyloid-like aggregates by yeast pyruvate kinase Cdc19, a central regulator of cellular metabolism and cell growth. Cdc19 forms aggregates under prolonged glucose starvation, which is reversed upon glucose supplementation [15]. This process protects Cdc19 from stress-induced degradation, thereby ensuring the restart of the cell cycle after stress [15]. Secondly, reversible aggregates are commonly associated with protein storage [8,11,42,43]. For instance, prolactin and growth hormone (GH) are stored in concentrated forms as protein aggregates in secretory granules. When needed, the protein aggregates are rapidly dissolved into monomers, causing a hormone burst in the bloodstream [8,43]. Thirdly, reversible regulation plays a vital role in the clearance of condensates, as well as the degradation and clearance of disease-associated amyloid aggregates [44]. For clearance and degradation, several pathways and machineries exist, e.g., autophagy, the ubiquitin-proteasome system (UPS), molecular chaperones, and protein disaggregases [45]. The accumulation of aberrant aggregates can be caused by proteostasis impairment, together with the appearance of the corresponding diseases [46,47]. Autophagy is an essential degradation pathway in clearing aberrant aggregates, such as those formed by TDP-43, α-synuclein, tau and Aβ [48,49,50,51]. Impairments in autophagy are strongly associated with neurodegeneration, such as Alzheimer’s disease (AD) and Parkinson’s disease (PD) [52,53]. Compounds that stimulate autophagy have been shown to improve TDP-43 clearance and to prevent TDP-43 mediated cell death in a neuronal model with amyotrophic lateral sclerosis (ALS) [48]. UPS is another pathway for protein degradation [54], and impairment in UPS is also implicated in neurodegeneration [55]. The accumulation of insoluble tau is associated with the dysfunction of the proteasome and inhibition of ATPase in a mouse model. Increased cAMP concentrations in the brain can enhance the activity of the proteasome and thereby promote the degradation of tau aggregates [55]. In the cell, machineries such as molecular chaperones and proteases are required for functional protein disaggregation. Upon heat stress, Pub1 forms biological condensates that are related to a restart of the cell cycle [56]. Release of the cell cycle arrest coincides with condensate dissolution. In vitro, heat shock proteins (HSPs) such as Hsp104 are required for the disassembly of heat-shock induced condensates [56]. Moreover, HSPs and protein disaggregases are also implicated in combating protein misfolding and aberrant protein aggregation [57,58]. HSPs (e.g., Hsp70 and Hsp90) are important inhibitors of protein aggregation by protecting the exposed hydrophobic regions from aggregation [59,60,61]. Yeast Hsp104 can reverse toxic aggregates formed by α-synuclein, tau, Aβ, PrP and amylin [62,63], thus reducing the toxic species and restoring native function to proteins sequestered within aggregates [59]. Engineered Hsp104 variants have an enhanced ability to dissolve protein aggregates that are associated with neurodegenerative diseases such as PD and ALS [64,65]. Altogether, these observations indicate that HSPs and proteases are used by cells to effectively reverse both functional and aberrant aggregates, in order to maintain cellular function and homeostasis.

## 3. Other Factors Regulating Functional Protein Aggregation

Except rapid growth rates and reversible dynamics, there are several other ways to avoid potential toxicity by regulating functional aggregation [32]. The expression and degradation of the precursors of functional amyloid fibrils are tightly controlled because high levels of an amyloidogenic precursor could initiate unwanted amyloid aggregation [32]. Moreover, in order to prevent unexpected interactions between aggregates and other cellular components, numerous functional protein aggregation reactions occur within distinct compartments, e.g., melanosomes, endocrine granules and acrosomes [7,32,42,66]. Finally, diverse classes of biomolecules have been identified in regulating functional protein aggregation to ensure correct structural assembly and/or disassembly, e.g., lipids/membranes, glycosaminoglycans (GAGs), nucleic acids, metal ions, heat shock proteins and proteases. A full understanding of how these molecules and other biochemical factors regulate functional protein aggregation is only just starting to emerge, and will be briefly discussed hereafter.

### 3.1. Polyanions

Polyanions, e.g., membranes containing negatively charged lipids, GAGs and nucleic acids, can act as effective catalysts for protein aggregation by providing a platform where proteins bind through electrostatic interactions, thus enhancing the local protein concentration. Through these interactions, monomers can adopt a conformation and/or orientation that promotes their assembly into fibrillar structures [67,68,69,70,71,72,73].

#### 3.1.1. Lipids/Membranes

Membranes are involved in the amyloidogenesis of several proteins, such as Aβ, prion protein (PrP), α-synuclein, islet amyloid polypeptide (IAPP), and Orb2A [74,75,76]. Upon interaction with membranes, proteins can undergo a series of conformational changes, thus inducing the formation of oligomers that are rich in cross β-sheet structures (annular pores and amyloid fibrils) [75]. Membrane binding can also be directly involved in regulating functional amyloid formation [76,77]. For example, Orb2 binding to anionic membranes results in a transition from a dynamic, intrinsically disordered state to a less dynamic *α*-helix which prevents β-sheet formation and amyloidogenic aggregation of Orb2 [76]. This inhibition by anionic membranes is proposed to be a potential mechanism regulating Orb2 amyloidogenesis in vivo. A similar mechanism has been identified for aggregate formation of Pmel17, involved in enhancing melanin synthesis [66]. Aggregation of the repeat domain (RPT) derived from Pmel17 is modulated by lysophospholipid-containing vesicles [77]. The surfactant-like lysophospholipid is of particular interest due to its high content in melanosomal membranes, and it has been suggested that protein-lysophospholipid interactions within melanosomes may regulate functional aggregation of Pmel17 in vivo [77]. TasA, a major matrix protein in biofilms of *Bacillus subtilis*, interacts distinctively with bacterial model membranes. In the presence of eukaryotic model membranes or in the absence of membranes, TasA forms fibers of similar structure and morphology. However, upon the interaction of TasA with bacterial model membranes, disordered aggregates with a different β-sheet signature are formed and bacterial membranes deformed more extensively than eukaryotic membranes, which could be crucial in providing integrity to biofilms [78]. Of note, regulation of amyloid formation by membranes must be carefully regulated, as oligomers or aggregates formed on membranes can cause damage to membranes, which is considered to be a main mechanism of amyloid toxicity [79].

#### 3.1.2. GAGs

Glycosaminoglycans (GAGs) are long, unbranched polysaccharides consisting of repeating disaccharide subunits. They exist on the surface of cells or in the extracellular matrix of multicellular organisms. GAGs, particularly heparan sulfate (HS) and its highly sulfated derivative heparin, play important roles in protein aggregation, stability and resistance to proteolysis [69]. Pathological aggregates of Aβ42, tau, α-synuclein and PrP induced by GAGs are implicated in neurodegenerative diseases [80]. GAGs are also able to reduce the cytotoxicity of a number of amyloid systems by different mechanisms [81]. GAGs-accelerated aggregation can provide protection against the cytotoxicity of intermediate oligomers. For instance, heparin enhances the fibrillogenesis of IAPP aggregation that is associated with type 2 diabetes. Heparin inhibits IAPP cytotoxicity in islet cells, whereas in GAG-deficient cell lines, IAPP-induced toxicity could not be prevented [82]. Alternatively, protein interaction with GAGs can increase protein stability and decrease its propensity to aggregate, such as in the case of PrP [83]. The exact mechanism by which different GAGs influence protein structure and aggregation, as well as the intercellular spread of these aggregates, remains elusive. Subtle differences in the GAG backbone structure and charge density significantly alter the properties of the resulting amyloid fibrils, as was shown for α-synuclein [84]. Distinct fibrils displayed variable levels of cytotoxicity, but also exhibited an altered ability to internalize into cells [84]. Finally, GAGs can inhibit the toxicity of protein oligomers by binding the oligomers to the cell surface, and in this way preventing the interaction of the oligomers with cells, as has been shown for *Escherichia coli* HypF (HypF-N) [85]. GAGs can also play a role in enhancing functional protein oligomerization and aggregation. For example, low molecular weight heparin is able to induce many hormone peptides or proteins to form amyloid fibrils [7].

#### 3.1.3. Nucleic Acids

Nucleic acids (DNA and RNA) are large polymers of nucleotides with characteristics of polyanions. Nucleic acids can not only induce and accelerate protein aggregation of e.g., PrP, α-synuclein, amyloid-β and huntingtin [72], but can also reverse protein aggregation [86]. For example, HIV-1 Gag protein undergoes nucleic acid-dependent aggregation, and an excess of nucleic acids can promote the disassembly of the formed aggregates [86]. Additionally, nucleic acid-bound proteins are common components of membraneless compartments (e.g., nucleoli, germ granules, Cajal bodies, stress granules (SGs) and P-bodies) that exhibit liquid-like properties [17,87,88,89,90,91,92,93,94,95]. These cellular compartments are associated with diverse biological processes, including RNA metabolism, ribosome biogenesis, DNA damage response and signal transduction [87]. Their components are highly mobile and can exchange with the surrounding medium rapidly and specifically through protein assembly and disassembly [96,97].

#### 3.1.4. Polyphosphate (PolyP)

PolyP contains a linear arrangement of inorganic phosphates that are connected via phosphoanhydride bonds [98]. PolyP has been indicated to exhibit both anti- and pro-aggregation properties [99]. Specifically, under oxidative stress or heat shock condition, polyP acts as a protein-stabilizing scaffold that binds to protein unfolding intermediates and stabilizes them in a soluble β-sheet-rich conformation, which prevents protein aggregation. Once the stress is released, the polyP-bound proteins are refolded and restored into their native structures. PolyP is able to enhance both functional amyloid formation (e.g., bacterial CsgA [100]) and disease-related amyloid aggregation (e.g., Aβ, α-synuclein and tau), and can also cause morphological changes in mature fibrils [100,101]. In general, however, amyloid aggregates formed in the presence of polyP are not cytotoxic, and therefore polyP is proposed as a physiologically relevant modifier in amyloidogenic processes [99].

### 3.2. Metal Ions

Metal ions participate in both pathological and functional protein oligomerization and aggregation via a variety of different mechanisms. Metal ions can (1) bridge two peptides or proteins; (2) change the overall charge of proteins; (3) induce a conformational change within a protein; (4) induce changes in fiber morphology [102].

Metal ions, such as Fe^3+^, Cu^2+^ and Zn^2+^, play essential roles in the brain. Toxic exposure of the brain to metals and/or dyshomeostasis in metal metabolism are associated with protein misfolding and aggregation in neurodegenerative diseases [103,104]. Specifically, Zn^2+^ ions accelerate the aggregation of both Aβ and tau, which are the hallmarks of AD. High concentrations of Zn^2+^ binding to Aβ causes an immediate conformational transition to a hydrophobic state which promotes fast protein aggregation [105]. Zinc ions also enhance tau aggregation and accelerate tau toxicity in neuronal cells via inducing the formation of intermolecular disulfide bonds [106]. Metal ions are also involved in regulating functional protein oligomerization and aggregation. For example, Ca^2+^ binding to the EF-hand motifs of S100A12 and Zn^2+^ binding to the dimeric S100A12 interface cooperatively induce a conformational rearrangement within the protein that leads to protein oligomerization, which plays a role in responding to inflammation [107,108]. Zinc ions also induce growth hormone aggregation, which facilitates peptide storage [8]. Potassium channel tetramerization domain containing 1 (KCTD1) family proteins play a role in regulating different signaling pathways. Copper ions binding to KCTD1 results in increased β-sheet content, promoting amyloid aggregation that is functionally cytotoxic in initiating apoptosis [109].

### 3.3. Post-Translational Modifications

Most proteins translated from mRNA are subject to post-translational modifications (PTMs) before executing their function(s) in different cell types. PTMs play a vital role in generating protein heterogeneity and utilizing identical proteins for different cellular functions in different cell types. PTMs such as methylation [110], glycosylation [111], acetylation [112,113,114,115], phosphorylation [15,25,116,117,118,119,120,121,122] and cysteine modification [123,124,125,126,127,128,129,130] are major factors in modulating protein self-assembly and disassembly. Phosphorylation is proposed as a protective mechanism to reduce toxic protein aggregation [118,120]. For instance, hyperphosphorylation of the C-terminus of TDP-43 favors its dissociation from aggregation and may facilitate its degradation by UPS [120]. Phosphorylation and other PTMs are suggested as generic mechanisms to reversibly regulate the aggregation of proteins containing low complexity regions (LCRs) [15,25]. For example, phosphorylation and dephosphorylation of the hydrophobic, aggregation-prone LCR region in Cdc19 prevents and enhances protein aggregation, respectively [25]. Crosstalk between different PTMs provides an additional layer of regulation of protein aggregation [131,132,133]. This is illustrated by the fact that phosphorylation of mutant huntingtin exon1 (Httex1) inhibits protein aggregation, whereas acetylation reverses the inhibitory effect [132].

Cysteine oxidation is another well-studied PTM, playing a significant role in the regulation of protein structure, stability, oligomerization and function [123,124,134]. The thiol group of cysteine is sensitive to redox conversion. Cysteine residues can be reversibly oxidized to a disulfide bond and to sulfenic acid, or irreversibly oxidized to sulfinic acid and sulfonic acid [135,136]. Sulfenic acid modification of Cys-111 triggers the formation of nascent superoxide dismutase 1 (SOD1) oligomers, which induce the fibrillization of both SOD1 and TDP-43 in cells [137]. Sulfonic acid modification of a single cysteine in FF domain, a conserved domain involved in transcription, RNA splicing and signal transduction [138,139], suffices to enhance protein aggregation through destabilization of the native conformation [124].

Transition between free cysteines and disulfide bonds is the most commonly occurring reversible redox reaction involving cysteines, and enables the flexible regulation of protein structure and function [140]. Native disulfide bonds are crucial for protein stability [141], and their disruption can lead to protein destabilization and aberrant aggregation, as has been described for e.g., SOD1, amylin and PrP [125,127,142,143]. For example, the disulfide bond in the amylin monomer stabilizes the N-terminal α-helical structure, which prevents the formation of β-sheet structures [144,145]. The disulfide loop also protects amylin from aggregation through binding to the amyloid-prone regions of amylin monomers [146]. The removal of disulfide bonds in native amylin oligomers causes structural changes, decreases polymorphism and induces protein aggregation [125].

Disulfide bond cleavage also enhances functional protein aggregation. An interesting example is presented by premelanosome protein (PMEL). PMEL forms a disulfide-bonded homodimer which involves a cysteine-rich Kringle-like domain (KLD). This KLD is required to resolve PMEL dimers that are formed in the endoplasmic reticulum into monomeric forms within the late Golgi or a post-Golgi compartment. The cysteine residues within this KLD initiate a disulfide exchange between intermolecular disulfide bonds (between PMEL monomers) to intramolecular disulfide bonds (within a PMEL monomer) in an autocatalytic manner [147]. Somatostatin-14 (SST-14) is a cyclic peptide hormone, and an amyloid structure is implicated in its storage. The disulfide bond in SST-14 controls the cyclization process and hence its conformational flexibility, which in turn associates strongly with its aggregation and disaggregation profiles. Native SST-14 aggregation needs prolonged incubation and the resulting amyloids readily release the monomers. In contrast, cleavage of the disulfide bond results in noncyclic SST-14, which may lead to increased accessibility to the aggregation-prone region and heparin-interaction ability. As a result, the self-association capacity of SST-14 is enhanced, but with a slower monomer releasing potency [126]. These results also indicate a marked variation in the interpeptide hydrogen bonding network upon cleavage of the disulfide bridge. Formation of non-native intermolecular disulfide bonds are also associated with protein aggregation [128,129,148,149,150]. For instance, upon necroptosis induction, aggregates of receptor-interacting protein kinase 1 and 3 (RIPK1/RIPK3) concentrate and phosphorylate MLKL, inducing structural changes of MLKL and facilitating disulfide bond formation. Disulfide bonds stabilize the adopted structure and enhance downstream amyloid aggregation of MLKL, which is functional in necroptosis [13]. Another example is represented by p16^INK4A^, which forms disulfide bridged homodimers under mild oxidizing conditions. This dimerization induces conformational rearrangements and leads to amyloid aggregation. The accumulation of p16^INK4A^ inhibits oncogenic transformation through regulating cell cycle arrest and senescence [130].

### 3.4. Emerging Factors Affecting Protein Aggregation

The presence of amyloid aggregates in the acrosomal matrix (AM) contributes to the stability of the AM core, which plays an important role in sperm-zona pellucida (ZP) interactions. During the acrosome reaction, the reversal of amyloid aggregates has been suggested to be an integral part of AM dispersion [42]. Active proteases are suggested to be responsible for subsequent AM disassembly [151]. These results suggest that (glyco)protein assemblies could also function as a surface to stimulate protein aggregation, possibly by polyanionic interactions.

Additionally, polyphenols can also affect protein aggregation at many levels. Polyphenols in combination with β-cyclodextrin (β-CD) inhibit and disaggregate α-synuclein aggregation [152]. The protective effect of polyphenols has also been indicated in breaking up the pathological aggregates formed by Aβ and tau [153]. Hydroxytyrosol is able to inhibit insulin amyloid formation and completely reverse the toxicity induced by amyloid insulin aggregates in the cell [154]. Therefore, polyphenols provide a promising approach for targeting neurodegenerative diseases.

In summary, protein aggregation within cells is a highly complex process that can be affected by multiple biomolecular factors, but a comprehensive view of how these factors individually and collectively contribute to this process is lacking. Difficulties to reveal clear mechanisms of the aggregation-related diseases is one of the reasons for a lack of efficient therapeutic strategies for these devastating diseases. It has been hypothesized that functional aggregates could be a precursor of pathological events [3]. Therefore, understanding how functional protein oligomerization and aggregation is regulated in the cell could be vital for elucidating the molecular and cellular factors promoting pathological aggregation, and for the design of new therapeutic strategies.

## 4. Crosstalk between Different Factors Affecting Amyloidogenesis of GAPR-1 and Members of the CAP Superfamily Proteins

Amyloid-like aggregation of GAPR-1 is mediated by a variety of biomolecular factors [155,156,157], as will be discussed below. We suggest that the cysteine-rich secretory proteins, antigen 5 and pathogenesis-related proteins group 1 (CAP) domain is a structural domain, which can utilize its amyloidogenic properties to regulate protein–protein interactions of other CAP family members as well, through distinct and controlled aggregation pathways.

### 4.1. Amyloid-Like Aggregation of GAPR-1

**Figure 2:**
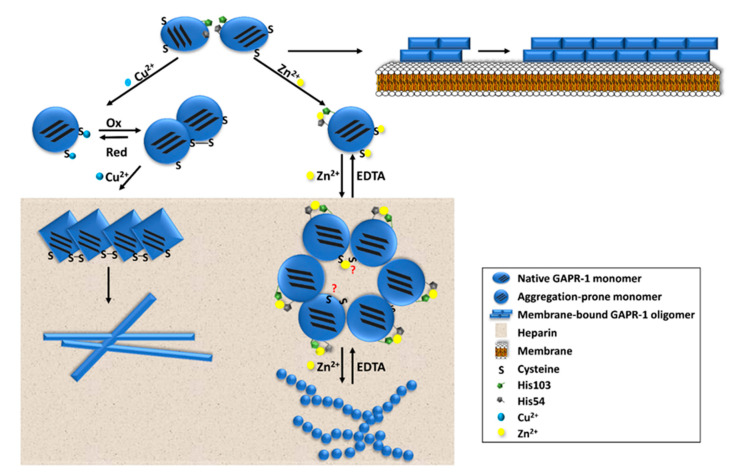
Hypothetical model for GAPR-1 amyloid-like aggregation. GAPR-1 binding to membranes undergoes a structural rearrangement and a concentration step, resulting in protein amyloid fibrillation [157,168]. Both zinc and copper ions binding modulate the quaternary structure of GAPR-1, shifting native multimers to monomers. Zn^2+^ induced aggregation pathway is dependent on the proposed metal binding site within GAPR-1, including His54 and His103, and independent of redox conditions. Zn^2+^ modulated GAPR-1 assembly is reversible by chelating zinc ions, which reversibly regulates the cysteine accessibility in GAPR-1. Disulfide bond formation is crucial for the initiation of Cu^2+^ induced aggregation pathway. Cu^2+^ regulated GAPR-1 self-association is irreversible and independent of the suggested metal binding site. Heparin acts as a scaffold on which GAPR-1 is concentrated and oriented to promote protein assembly [155,156].

GAPR-1 functions as a negative regulator of autophagy in mammalian cells [158]. It is associated with lipid-enriched microdomains at the cytosolic leaflet of Golgi membranes, where it inhibits autophagy by anchoring the autophagy-inducing protein Beclin 1 to the membrane [158,159]. How can these cell biological properties relate to the amyloidogenic behavior of GAPR-1? Two distinct aggregation pathways of GAPR-1 have been described [155,156,157], and the effects of zinc, copper, heparin, disulfide bond formation and membranes in these pathways are summarized in Figure 2.

Within each pathway, there are several characteristic elements of functional protein aggregation. Irrespective of the external trigger, GAPR-1 aggregation proceeds relatively fast, as determined with ThT fluorescence. This indicates that upon initiation, GAPR-1 exists only for a short time in a native-like oligomeric state. In the zinc-dependent pathway, GAPR-1 aggregation can be reversed by the depletion of the metal ion, whereas oxidative conditions promote fast, irreversible aggregation followed by formation of disulfide-bridged nuclei, resembling the classical nucleated growth mechanism.

The localization of GAPR-1 to lipid-enriched microdomains of the Golgi membrane allows locally increased concentrations of GAPR-1 and provides additional beneficial conditions for the formation of oligomeric structures. Tat-Beclin 1, a peptide derived from the evolutionary conserved domain (ECD) of Beclin 1 with high therapeutic potential, binds to GAPR-1 to release GAPR-1-bound Beclin 1 and to induce autophagy [158]. Interestingly, a similar mechanism was described in neuronal cells, where Beclin 1 was re-localized to lipid rafts of the plasma membrane by PrP to induce autophagy in response to Aβ [160]. The interaction between GAPR-1 and Tat-Beclin 1 is proposed to be tightly associated with the quaternary structure of GAPR-1 [161]. Beclin 1 is known to form homo-oligomers [162] and its ECD was shown to cluster upon membrane binding, resulting in the deformation of membrane surface areas [163]. We hypothesize that the interaction between GAPR-1 and Beclin 1 is dependent on their oligomeric/fibrillar states. In this light, the ability of GAPR-1 to interact with oligomeric Aβ [157] could suggest that GAPR-1 not merely keeps Beclin 1 inactive, but is capable of interacting with multiple oligomeric structures. In this model, GAPR-1 could function as a molecular sensor for multiple amyloid oligomers in the cell, assuming this interaction is based on structural properties. Thus, potentially harmful oligomers could successfully compete with Beclin 1 for an interaction with GAPR-1, resulting in the release of Beclin 1 and the subsequent activation of autophagy. We envision this mechanism of the cell to clear up potentially harmful oligomers, which could be a focus of future studies.

### 4.2. Potential Regulation of Amyloid-Like Aggregation of CAP Family Members

An analysis of structural determinants for the amyloidogenic propensity of GAPR-1 predicted amyloid-prone segments in the CAP1 and CAP2 motifs [157]. Due to the high conservation, CAP proteins from all taxa contain these potentially amyloidogenic segments within these signature motifs. This opens the possibility that a common function of the CAP domain lies within this structural property [157]. Several clues in literature provide support for this hypothesis.

Allurin, a truncated CRISP protein from the female tract of *Xenopus*, functions as a sperm chemoattractant [164]. Allurin was shown to be present in egg jelly (“egg water”) as stable, SDS- and 2-mercaptoethanol-resistant multimers [165]. Moreover, the oligomerization of rat CRISP1 was regulated by Zn^2+^ binding and crucial for its association to spermatozoa during epididymal maturation [166]. Similar to GAPR-1, human CRISP2 forms ThT-positive structures via interaction with PI-containing liposomes [157]. Natrin, a CRISP protein from snake venom, modulates inflammation via inducing the expression of vascular endothelial cell adhesion proteins [167]. This is proposed to involve heparan sulfate- and Zn^2+^-dependent dimerization and/or the oligomerization of natrin [167]. For a more extensive discussion, we refer to a recent review [168].

Protein oligomerization starts with dimerization, and a number of CAP family members possess this structural property. PR-1-type pathogenesis-related protein (PR-1-5) identified in wheat exists primarily as homodimers in vitro, and is resistant to proteases. Interestingly, a PR-1-5 monomeric mutant revealed a diminished protease resistance [169]. The dimeric PR-1-5 is able to interact with ToxA and is involved in ToxA-induced necrosis in sensitive wheat [170]. Other CAP family members that were shown to form dimers include Fpr1 from *Fusarium oxysporum* [171], and an *Ancylostoma*-secreted protein secreted by infective larvae of the human hookworm *Necator americanus* (*Na*-ASP-2) [172]). Several other CAP family members were shown to crystalize as stable dimers (e.g., an *Ancylostoma*-secreted protein from *Ostertagia ostertagi* (*Oo*-ASP-1) [173], *Na*-ASP-1 [174], hookworm platelet inhibitor (HPI) [175], a bacterial CAP superfamily protein (BB0689) [176] and natrin [177]). The crystal structure of a CAP superfamily protein from fungus (MpPR-1i) revealed a heptameric structure [178].

**Figure 3:**
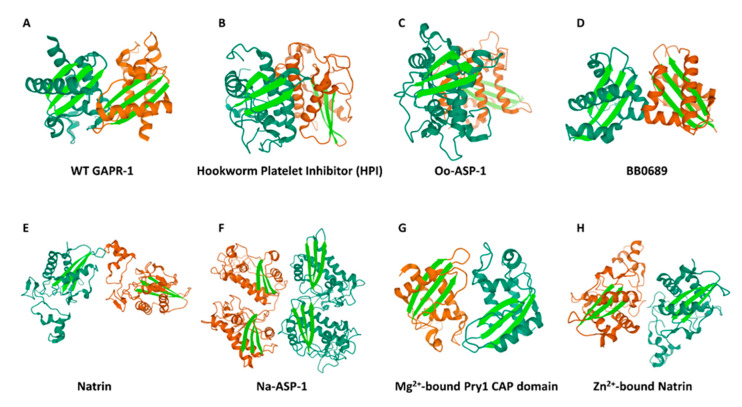
Tertiary structures of dimeric cysteine-rich secretory proteins, antigen 5 and pathogenesis-related proteins group 1 (CAP) superfamily proteins. The dimeric tertiary structures of GAPR-1 (**A**), Hookworn Paltelet Inhibitor (HPI) (**B**), Oo-ASP-1 (**C**), BB0689 (**D**), Natrin (**E**), Na-ASP-1 (**F**), Mg^2+^-bound Pry1 CAP domain (**G**) and Zn^2+^-bound Natrin (**H**) are presented with one monomer in dark green and another monomer in orange. β-Sheets are highlighted in light green. Images were created using 3D view in PDB website (www.rcsb.org) and the following PDB entry files: 1SMB (GAPR-1); 4TPV (Hookworn Paltelet Inhibitor, HPI); 4G2U (Oo-ASP-1); AD53 (BB0689); 2GIZ (Natrin); 3NT8 (Na-ASP-1), 5JYS (Mg^2+^-bound Pry1 CAP domain); and 3MZ8 (Zn^2+^-bound Natrin).

GAPR-1 is (partially) present as dimers in solution [179] and on Golgi membranes [159] and crystallizes as a dimer [180]. We recently proposed that the almost continuous β-sheet in crystallographic GAPR-1 dimers facilitates GAPR-1 oligomerization [168] (also see Figure 3A). This orientation of monomers in the dimer suggests that no dramatic structural rearrangements are required, and only subtle structural changes might be sufficient to allow an extension of the β-sheet structure in an oligomeric arrangement. Indeed, only minimal structural rearrangements are observed in the GAPR-1 molecule during amyloid-like aggregation [157].

An essential feature of the β-sheet continuation from one monomer to the next monomer is the antiparallel β-sheet arrangement of β-sheets in each monomer (Figure 3A). This arrangement of antiparallel β-sheets is present in all CAP family members, as part of the unique α-β-α-fold described in previously determined structures of the CAP superfamily [181]. Some of the CAP family members that crystallize as a dimer are shown in Figure 3. From these arrangements, it becomes clear that only some dimeric CAP family members show the formation of continuous β-sheets, such as the Mg^2+^-bound CAP domain of yeast Pry1 (Figure 3G) and the Zn^2+^-bound natrin dimer (Figure 3H) [167,182]. However, several other CAP family members do not form a continuous β-sheet in crystallographic dimers (Figure 3B–F). Nevertheless, in all cases, the β-sheets are readily exposed to the surface, and the formation of alternative dimeric arrangements could allow the formation of continuous β-sheets in dimeric/oligomeric structures. Thus, the formation of amyloid-like oligomeric structures would not depend so much on structural rearrangements in individual monomers (as indeed observed for GAPR-1), but rather depend on the formation of dimer structures with a continuous β-sheet arrangement. In this respect, it is interesting to note that GAPR-1 can form a different dimeric structure in the presence of IP6 [183].

Dimer formation may also induce different types of amyloid-like aggregation pathways. Indeed, the amyloid-like aggregation of GAPR-1 is controlled by the redox state and by different types of metal ions, allowing the control of different types of aggregation pathways (Figure 2), as visualized by electron microscopy [155].

Cysteine oxidation of monomeric GAPR-1 enhances the exposure of C-terminal aggregation-prone regions (APRs), which in turn accelerates GAPR-1 aggregation under oxidative conditions [155]. Whether the disulfide bond in the copper-induced aggregation pathway is formed intra- or inter-molecularly needs to be further explored. Different from GAPR-1, the majority of CAP superfamily members are secreted and contain significantly more cysteine residues, most of which are present in disulfide bonds. Disulfide bridges are responsible for protein stability in the extracellular environment, which is highly relevant for many CAP superfamily members. Specifically, disulfide bridges contribute strongly to the high thermal, pH, and proteolytic stability of PR-1-like proteins [184]. The absence of disulfide bridges in PR-1-like proteins in *Fusarium oxysporum* is proposed to render them more accessible to cleavage by host proteases, which may allow these types of fungi to evade detection by the plant immune system [171]. Moreover, *Oo*-ASP-1 from *Ostertagia ostertagi* forms inter-molecular disulfide-bond dependent dimers. The disulfide bond is important for both tertiary and quaternary structures of *Oo*-ASP-1, and is also suggested to be vital for proteolytic stability [173]. These evidences suggest that disulfide bonds and/or disulfide bridged oligomerization play crucial roles in the protein structural stability of CAP superfamily members, and in the regulation of their protein function.

Zinc and copper homeostasis can be a major factor in regulating distinct GAPR-1 aggregation pathways [155,156]. Zinc and copper are the second and third most abundant transition metals in organisms, respectively [185]. The amount of zinc and copper ions in cells (0.3–20 mM Zn^2+^, depending on cell type; <10^−18^ M Cu^2+^) and in blood (12–16 μM Zn^2+^; 10–22 μM Cu^2+^) is strictly maintained at low concentrations and most of it is associated with proteins [186,187,188]. Altered GAPR-1 aggregation is observed when copper ions are chelated by the addition of EDTA after a nucleation step in the presence of heparin and 20–100 μM copper [155]. This indicates that excess copper binds to GAPR-1 non-specifically, and that specific protein structural reorientations occur at low copper concentrations.

High concentrations of zinc are present in several organs and cell types, such as in the brain (up to 150 mM) [189], the mammalian testis, the epididymis, and in the prostate (1–2.5 mM) [186,190,191,192,193]. Zinc ions are widely associated with the reproductive process, including spermatogenesis, sperm maturation, capacitation, acrosome reaction, conception and embryonic implantation [194,195,196,197]. Cysteine-rich secretory protein 1 (CRISP1) is a member of the CAP superfamily; it is expressed in the epididymis and cumulus cells, and it plays multifunctional roles in spermatozoa maturation, capacitation, sperm-oocyte interaction and acrosome reaction [198,199,200,201,202,203,204]. Rat CRISP1 has been shown to oligomerize in the presence of 0.5–2 mM zinc ions in vitro, and these oligomers play a role in rat sperm maturation [166]. Our in vitro study shows that human CRISP1 is able to form high molecular weight structures in the presence of physiologically relevant zinc concentrations (0.3–1 mM), and that the oligomers are dissolved upon the removal of zinc ions (data not published). This indicates that, under physiological conditions, CRISP1 may exhibit its functions in fertilization through zinc regulated protein assembly and disassembly.

High expression levels of amyloidogenic proteins are also a key factor in amyloid formation [32] and CAP superfamily members are often found to be up-regulated under specific conditions. GAPR-1 is highly expressed in immune-related cells and tissues (e.g., monocytes, leukocytes, lung, spleen and embryonic tissue), and enriched in the lumen of the extracellular vesicles secreted from prostate cells [159,205]. GAPR-1 is also enriched during neonatally induced neurodegeneration in rat hippocampus [206]. Our combined results open the possibility that GAPR-1 oligomerization and aggregation may play a functional role in immunology, fertilization and autophagy. On the other hand, the level of oligomeric precursors of functional aggregates must be tightly regulated because the presence of high concentrations of amyloidogenic precursor could result in cellular disorders [32]. The expression level of GAPR-1 is significantly enhanced in the epithelial cells of fibrotic kidney [207]. These observations indicate that changes in GAPR-1 expression level are associated with regulating its biological function, which is one of the potential mechanisms to control functional aggregation. Many members of the PR-1 subfamily are up-regulated in the infected tissue [208,209]. At the structural level, PR-1 proteins contain a CAP domain with only short N- and C-terminal extensions, indicating that the properties of the CAP domain (e.g., oligomerization) determine the function of PR-1 proteins in plants. Dimerization has been shown to enhance the resistance of PR-1-5 to proteolytic attack, which may be involved in protease-mediated programmed cell death pathways in plants [169]. Moreover, CRISP3 is expressed at low levels in the normal prostate and is highly up-regulated in the cancerous prostate [210]. In contrast, glioma pathogenesis-related protein 1 (GLIPR1) is down-regulated in prostate cancer, while forced GLIPR1 overexpression is pro-apoptotic in prostate cancer cells and is suggested as a prostate-cancer therapy [211]. Altogether, these observations indicate that regulating the expression level of CAP superfamily members is a widely applied mechanism for regulating protein function. Whether these examples are related to the aggregation-related properties of CAP superfamily members remains to be investigated.

## Abbreviations

GAGs	glycosaminoglycans
PTMs	post-translational modifications
HSPs	heat shock proteins
polyP	polyphosphate
PMEL	pre-melanosomal protein
PG-1	protegrin-1
CRESs	cystatin-related epididymal spermatogenic proteins
PLD	prion-like domain
A-bodies	amyloid bodies
P-bodies	processing bodies
HSGs	heat-shock granules
SGs	stress granules
GH	growth hormone
AM	acrosomal matrix
USP	ubiquitin-proteasome system
RPT	repeat domain
HS	heparan sulfate
IAPP	islet amyloid polypeptide
AD	Alzheimer’s disease
PD	Parkinson’s disease
ALS	amyotrophic lateral sclerosis
HypF-N	Escherichia coli HypF
KCTD1	potassium channel tetramerization domain containing 1
LCR	low complexity region
Httex1	phosphorylation of mutant huntingtin exon1
SOD1	superoxide dismutase 1
PrP	prion protein
KLD	Kringle-like domain
SST-14	somatostatin-14
RIPK1	receptor-interacting protein kinase 1
MLKL	mixed-lineage kinase domain-like protein
ZP	sperm-zona pellucida
β-CD	β-cyclodextrin
GAPR-1	golgi-associated pathogenesis-related protein 1
CRISP 1	cysteine-rich secretory protein 1
ECD	evolutionary conserved domain
CAP	cysteine-rich secretory proteins, antigen 5 and pathogenesis-related proteins group 1
APRs	aggregation-prone regions
PR-1	pathogenesis related protein 1
GLIPR1	glioma pathogenesis-related protein 1
